# Factors Associated With Short and Long Term Cognitive Changes in Patients With Sepsis

**DOI:** 10.1038/s41598-018-22754-3

**Published:** 2018-03-14

**Authors:** Allan J. C. Calsavara, Priscila A. Costa, Vandack Nobre, Antonio L. Teixeira

**Affiliations:** 1 School of Medicine, Universidade Federal de Ouro Preto, Ouro Preto, MG Brazil; 2 Postgraduate Program in Health Sciences: Infectious Diseases and Tropical Medicine, School of Medicine, Universidade Federal de Minas Gerais, Belo Horizonte, MG Brazil; 3 Núcleo Interdisciplinar de Investigação em Medicina Intensiva - NIIMI, Belo Horizonte, MG Brazil; 4 Neuropsychiatry Program & Immuno-Psychiatry Lab, Department of Psychiatry and Behavioral Sciences, McGovern Medical School, University of Texas Health Science Center at Houston, Houston, TX USA

## Abstract

This study aimed to assess cognition in patients with severe sepsis or septic shock and whether cognitive impairment was associated with clinical and laboratory parameters. We conducted a cohort study of patients with severe sepsis and septic shock evaluated within 24 h and one year after ICU discharge. Demographic, clinical and laboratory data were analyzed, and the following neuropsychological tests were applied: Consortium to Establish Registry for Alzheimer’s Disease, Mini-Mental State Examination, and Trail Making Test forms A and B. We included 33 patients, mean age of 49, 19% were female. Patients underperformed on most measures 24 h after ICU discharge, with improvement on follow-up. IQCODE, APACHE II scores, NSE and IFN-γ levels at ICU discharge were associated with poor cognitive performance, while higher educational level was associated with good cognitive performance. The time to first antibiotic dose, accumulated dose of haloperidol during UCI stay and mean glycemia were also associated with poor cognitive outcome. In general, patients with severe sepsis or septic shock have cognitive impairment that can improve over time. This improvement was associated with factors identified during their ICU stay, such as cognitive reserve, educational level, mean glycemia during ICU stay and NSE level.

## Introduction

It is estimated that annually 31.5 million people have sepsis and 19.4 million have severe sepsis, with a hospital mortality of 17% and 26%, respectively^1^. Among survivors, a high percentage experiences physical and psychological sequelae, including cognitive impairment^2^. On May 2017, the Word Health Organization (WHO) recognized sepsis as a Global Health Priority and was committed to improve the prevention, diagnosis, and management of sepsis^3^.

Septic associated encephalopathy (SAE) is a condition characterized by cognitive dysfunction due to sepsis, without the presence of infection in the central nervous system or structural brain injury after excluding metabolic causes. This cognitive dysfunction is defined by new or exacerbation of preexisting deficits in global cognition or executive function^4^.

In a recent systematic review, we found that several factors are implicated in the occurrence of cognitive impairment in the context of sepsis^5^. Pre-sepsis depressive symptoms, number of hospital visits due to infection, temporal proximity to the latest episode of sepsis, length of hospitalization, among others, have been proved to be risk factors for SAE^6,7^. Conversely, length of stay in the intensive care unit (ICU), number of days on mechanical ventilation, APACHE II, and SOFA scores, patient’s age, family history of psychiatric illness, and substance abuse were among factors that were not shown to be associated with cognitive impairment after sepsis^7,8^.

To the best of our knowledge, no study has been specifically designed to assess the risk factors associated with the development of post-sepsis cognitive impairment^5^. It is worth mentioning that there is no consensus regarding the most appropriate neuropsychological tests for identification and/or follow-up of SAE.

In this context, this study aimed at comprehensively describing the characteristics of cognitive impairment in patients after severe sepsis or septic shock and to explore factors potentially associated with it in the short and long terms. The Consortium to Establish a Registry for Alzheimer’s Disease (CERAD), as a measure of global cognition, and the Trail Making Test (TMT), as a measure of executive function, were used to provide reliable and valid assessment of cognitive changes after sepsis. Besides clinical measures, inflammatory and neuronal-related biomarkers were measured due to their potential role in cognition.

We hypothesized that factors associated with the severity of the sepsis (e.g. APACHE, dose of certain drugs such as noradrenaline, requirement of mechanical ventilation and hemodialysis) could contribute to different weights in cognitive performance of septic patients.

## Results

### Demographic and clinical characteristics

**Figure 1:**
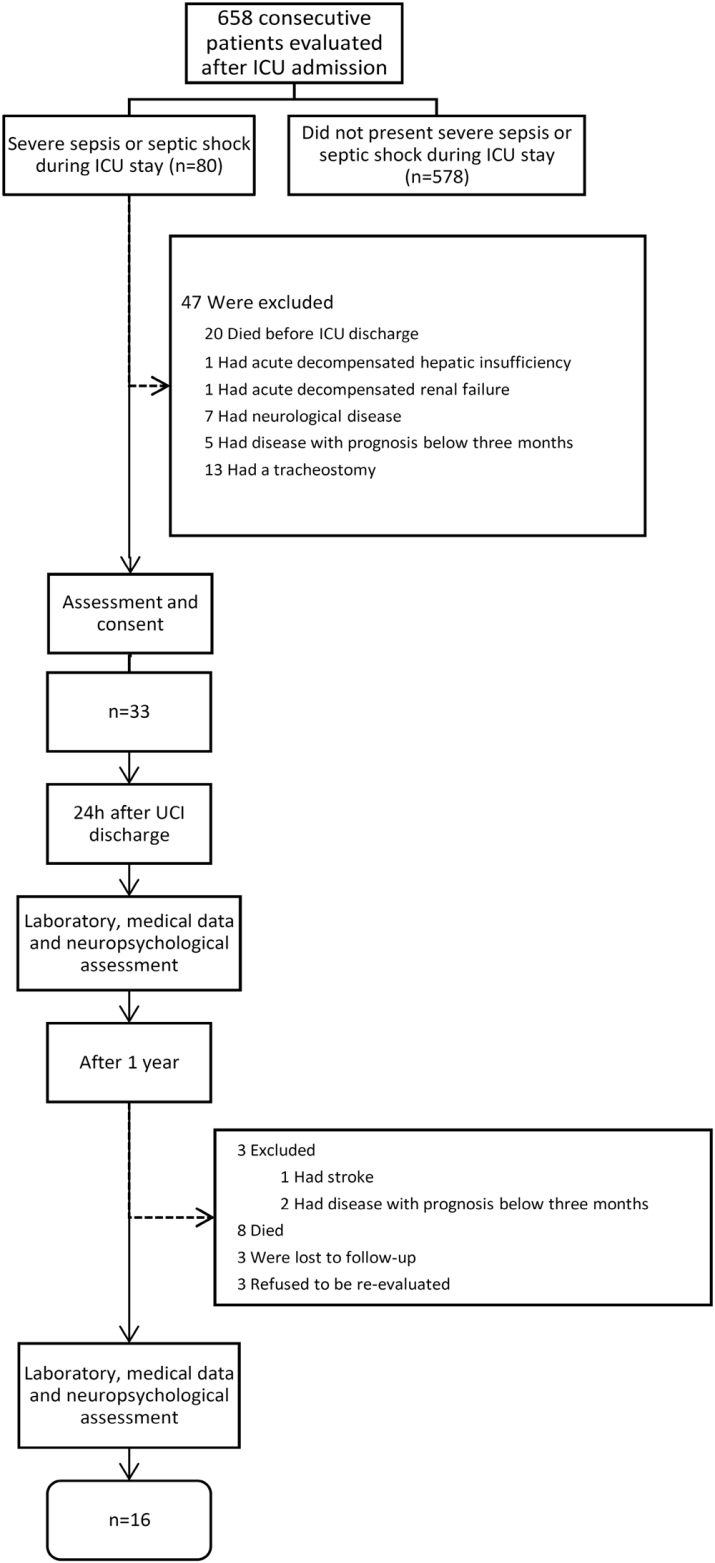
Flowchart detailing inclusion and exclusion of patients and protocol of study.

Of the 658 consecutive patients admitted in ICU, a total of 80 patients had severe sepsis or septic shock during the study period, 33 patients were initially identified as eligible and were evaluated within 24 h after ICU discharge. Sixteen of them (48%) were re-evaluated around one year of discharge [median 393 days]. The reasons for non-inclusion are reported in Fig. 1, and included: (1) absence of severe sepsis or septic shock during ICU stay, (2) death before ICU discharge, (3) tracheostomy, (4) prior or current neurological diseases. Comparison of demographic and clinical characteristics between patients re-evaluated and not re-evaluated in one year follow-up showed that these groups do not present significant differences in their baseline characteristics (see Supplementary Table. S1).

The main clinical and demographic characteristics of the included patients are shown in Table 1. The median APACHE II score of patients included in the study was 16 (12.5–20.5). Most patients had septic shock (75.8%) and in one third of them the site of infection was the lower respiratory tract.

**Table 1:** Clinical and demographic characteristics of patients included in the study (n = 33).

Age at sepsis (years)	49.0 ± 15.2
Female	19 (57.6)
Education (years)	7 (4–8)
APACHE II Score	16.0 (12.5–20.5)
SOFA Score at ICU admission	6 (5–9)
Administration of antimicrobial after recognition of septic shock or severe sepsis (hours)	2.5 (1.4–8.2)
ICU length of stay (days)	6 (4–13)
Laboratory at admission in ICU	
HGB (g/dL)	9.7 (8.7–11.2)
Creatinine (mg/dL)	0.9 (0.6–1.8)
Lactate (mmol/L)	1.9 (1.0–2.6)
CRP (mg/dL)	232.5 ± 128.5
Mean blood glucose during stay in ICU (mg/dL)	135.0 ± 36.7
Septic shock	25 (75.8)
Sites of infection	
Lung	11 (33.3)
Intra-abdominal	6 (18.2)
Urinary tract	5 (15.2)
Catheter	5 (15.2)
Skin	2 (6.1)
Unknown	4 (12.1)
Positive blood culture	13 (39.4)
Comorbid diseases	
Congestive heart failure	3 (9.1)
Chronic renal failure	6 (18.2)
Arterial hypertension	15 (48.5)
Diabetes mellitus	9 (28.1)
IQCODE	3.10 (3.00–3.47)
Cumulative dose	
Midazolam (mg)	180.0 (95.0–1130)
Fentanyl (μg)	3170.0 (620.0–9992.5)
Noradrenaline (mg)	35.5 (10.0–62.0)
Dobutamine (mg)	1679.2 ± 1416.3
Haloperidol (mg)	7.5 (2.5–26.7)
Need for corticosteroids in the first 72 h	6 (18.0)
Need for mechanical ventilation in the first 72 h	20 (60.6)
Need for hemodialysis in the first 72 h	3 (9.1)

Data presented as mean ± SD, N (%) or median (IQR_25–__75_).

Among 17 patients that were not re-evaluated in one year, five died during hospitalization, three died after hospital discharge, three presented exclusion criteria at the time of re-evaluation, three refused to participate and three were not localized (Fig. 1).

### Cognitive performance

In comparison with normative data, patients underperformed on MMSE and constructional praxis 24 h after ICU discharge, and on constructional praxis one year after discharge (Table 2). As expected, there was a significant increase in the score of several subtests one year after UCI discharge, the exceptions being the Boston Naming test and the constructional praxis test.

**Table 2:** Mean score obtained in CERAD battery subtests 24 h after discharge from ICU and after 1 year. The means were compared with expected cutoff point at each evaluation and between the cognitive assessments.

	24 h after ICU discharge^1^					1 year after ICU discharge^1^					24 h vs. 1 year^2^	
	n	M	SD	t	p	n	M	SD	t	p	t	p
Verbal Fluency	33	9.79	4.26	−1.63	0.056	16	12.88	4.32	1.74	0.948	**−2**.**36**	**0**.**025**
Modified Boston Naming Test	33	11.12	2.98	−1.70	0.05	16	11.56	1.97	−0.89	0.193	−0.54	0.594
MMSE	**33**	**21**.**21**	**4**.**70**	**−5**.**85**	**<0**.**001**	16	25.00	2.66	−1.51	0.077	**−3**.**59**	**0**.**001**
Word List Learning	33	13.55	4.81	0.65	0.742	16	18.00	4.08	4.90	1.000	**−3**.**19**	**0**.**003**
Word List Recall	33	3.27	2.67	0.59	0.717	16	4.94	2.26	3.42	0.998	**−2**.**14**	**0**.**037**
Word List Recognition Discriminability	33	6.45	3.40	−0.92	0.180	16	8.50	1.67	3.59	0.999	**−2**.**82**	**0**.**007**
Constructional praxis	**33**	**6**.**33**	**2**.**87**	**−5**.**34**	**<0**.**001**	**16**	**7**.**06**	**1**.**48**	**−5**.**23**	**<0**.**001**	−0.95	0.345
Praxis Recall	33	3.45	2.66	−1.18	0.122	16	5.31	2.68	1.96	0.965	**−2**.**29**	**0**.**027**

^1^One-Sample t-Test, one-tailed probability P (Hypothesized mean > sample mean).

^2^Independent-Samples t-Test. M = median, SD = standard deviation.

In addition to the CERAD battery, the TMT forms A and B were applied (Table 3). A considerable number of patients could not even finish the TMT before the maximum time of five minutes traditionally allotted for the task. Although there was a significant reduction in the average time required in the tests one year after discharge, just one patient had a normal score in the TMT form A in the re-evaluation. Similarly, no patient could run the TMT form B within the expected time either 24 hours after discharge from the ICU or in the revaluation.

**Table 3:** Performance of patients on Trial Making Test Form A and B.

	24 h after ICU discharge	1 year after ICU discharge	p-value 24 h vs. 1 year
**Trail Making Test A**			
Patients who required less than 300 seconds to complete the test N (%)	21 (63.6)	13 (81.3%)	0.324
Time to complete trail (s)	157.00 (98,5–276,8)	74.0 (56,5–150,5)	0.007
Not impairment time or error score, N (%)	1 (3.8)	1 (6.3)	1.000
**Trail Making Test B**			
Patients who required less than 300 seconds to complete the test N (%)	9 (27.3)	5 (31.2)	1.000
Time to complete trail (s)	228.5 (117.0–300.0)	110.5 (90,0–182.0)	0.009
Not impairment time or error score, N (%)	0 (0.0)	0 (0.0)	

We performed marginal regressions for the total score of the CERAD battery and for the MMSE. The models could explain 52.2% of the CERAD variance and 44.9% of the MMSE variance (Tables 4 and 5).

**Table 4:** Association between selected variables and CERAD total score in patients with severe sepsis and septic shock.

Variable	β	SE (β)	O.R.	C.I. 95%	p-Value
Intercept	4.128	0.170	—	—	0.000
IQCODE	−0.112	0.048	0.89	[0.81–0.98]	0.020
NSE at discharge/10	−0.091	0.037	0.91	[0.85–0.98]	0.013
IFN-γ at discharge/10	−0.302	0.041	0.74	[0.68–0.80]	0.000
Education, years/10	0.222	0.048	1.25	[1.14–1.37]	0.000
APACHE II	−0.011	0.004	0.99	[0.98–0.99]	0.005
Re-evaluation about 1 year	0.260	0.052	1.30	[1.17–1.44]	0.000

R² = 52.2%; Large VIF = 1.82. SE: standard error, OR: odds ratio, C.I. confidence interval, NSE: neuron specific enolase.

**Table 5:** Association between selected variables and MMSE score in patients with severe sepsis and septic shock.

Variable	β	S.E. (β)	O.R.	C.I. 95%	p-Value
Intercept	2.995	0.094	—	—	0.000
IL-6 at discharge /100	0.075	0.019	1.08	[1.04–1.12]	0.000
Education, years /10	0.089	0.020	1.09	[1.05–1.14]	0.000
Administration of antimicrobial after recognition of septic shock or severe sepsis, hours	−0.041	0.006	0.96	[0.95–0.97]	0.000
Haloperidol (Cumulative dose)/10	−0.032	0.015	0.97	[0.94–0.99]	0.034
Mean blood glucose during ICU stay (mg/dL)/10	−0.014	0.007	0.99	[0.97–0.99]	0.049
Re-evaluation about 1 year	0.173	0.046	1.19	[1.09–1.30]	0.000

R² = 44.9%; Large VIF = 1.23. SE: standard error, OR: odds ratio, C.I. confidence interval, ICU: intensive care unit.

Patients presented in the second evaluation a mean ± SD CERAD total score of 62.9 ± 8.9, 30% higher than in the first assessment. IQCODE, APACHE II score, NSE and IFN-γ serum levels at ICU discharge were associated with poor cognitive performance, while higher educational level was associated with good cognitive performance (Table 4).

Patients presented in the second evaluation a mean ± SD MMSE score of 25 ± 2.7, 19% higher than in the first assessment. The interval from recognition of severe sepsis or septic shock to the initial administration of antibiotic, accumulated dose of haloperidol during ICU stay and mean blood glucose throughout the ICU stay were associated with poor cognitive performance, while IL-6 on discharge and educational level were associated with good cognitive performance (Table 5).

### Exploratory analysis for biomarkers

**Figure 2:**
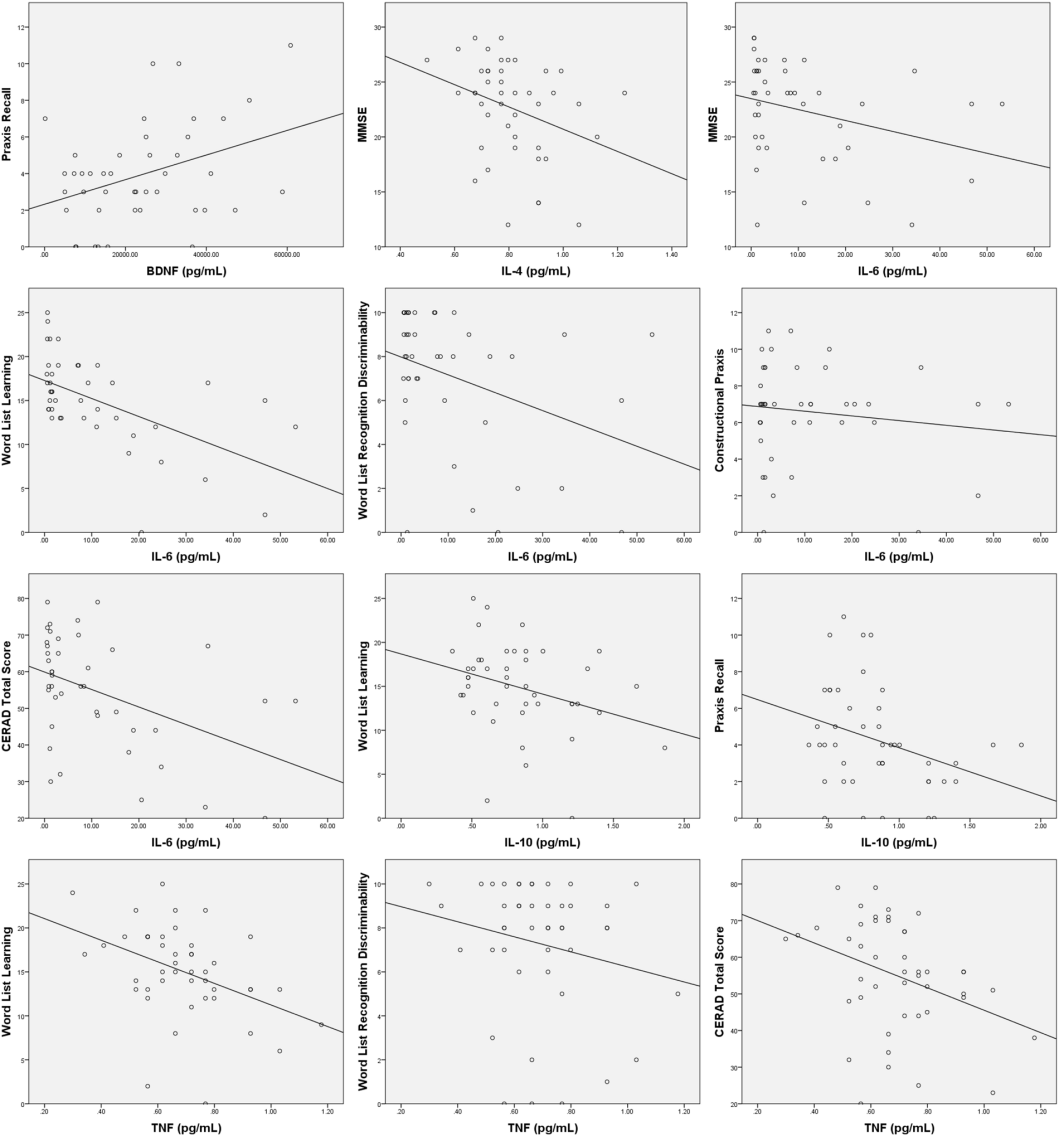
Correlation between serum cytokine level and CERAD subtests/total score at ICU discharge.

Correlational analysis was used to explore the possibly of linear correlation between biomarkers and cognitive performance evaluated by the CERAD battery (Table 6). BDNF correlated positively with praxis recall. Negative correlations were found between IL-4 and MMSE, IL-6 and MMSE, word list learning, word list recognition discriminability, constructional praxis, and CERAD total score, IL-10 and word list learning and praxis recall, TNF and word list recognition discriminability, CERAD total score and word list learning (Fig. 2).

**Table 6:** Bivariate correlation between biomarkers (BDNF, NSE, sTREM-1, IL-2, IL-4, IL-6, IL-10, TNF, IFN-γ and IL-17A) and CERAD subtests / total score in patients with severe sepsis and septic shock.

		VF	MBNT	MMSE	WLL	WLR	WLRD	CP	PR	CTS
BDNF	***r***	0.098	0.167	0.199	0.209	0.065	0.213	0.215	**0**.**370**	0.222
	***p***	0.533	0.284	0.200	0.180	0.678	0.171	0.166	**0**.015*	0.153
	***n***	43	43	43	43	43	43	43	**43**	43
NSE	***r***	−0.094	0.077	−0.079	0.252	0.113	0.006	−0.036	0.125	0.083
	***p***	0.545	0.617	0.611	0.099	0.466	0.967	0.818	0.419	0.590
	***n***	44	44	44	44	44	44	44	44	44
sTREM-1	***r***	−0.182	−0.028	−0.061	−0.088	−0.074	−0.041	0.053	−0.255	−0.099
	***p***	0.230	0.853	0.690	0.567	0.629	0.787	0.729	0.091	0.517
	***n***	45	45	45	45	45	45	45	45	45
IL-2	***r***	−0.064	−0.063	0.136	−0.086	0.106	−0.068	0.045	−0.072	−0.048
	***p***	0.692	0.693	0.397	0.591	0.511	0.670	0.781	0.656	0.767
	***n***	41	41	41	41	41	41	41	41	41
IL-4	***r***	−0.254	−0.225	**−0**.**324**	−0.246	−0.074	−0.234	−0.196	−0.183	−0.293
	***p***	0.096	0.142	**0**.032*	0.107	0.635	0.126	0.202	0.234	0.054
	***n***	44	44	**44**	44	44	44	44	44	44
IL-6	***r***	−0.274	−0.014	**−0**.**311**	**−0**.**560**	−0.135	**−0**.**365**	**−0**.**371**	−0.116	**−0**.**439**
	***p***	0.079	0.928	**0**.045*	**0**.000*	0.395	**0**.017*	**0**.016*	0.463	**0**.004*
	***n***	42	42	**42**	**42**	42	**42**	**42**	42	**42**
IL-10	***r***	−0.160	0.108	−0.042	**−0**.**316**	−0.067	−0.103	−0.161	**−0**.**327**	−0.200
	***p***	0.299	0.486	0.785	**0**.037*	0.664	0.506	0.296	**0**.030*	0.194
	***n***	44	44	44	**44**	44	44	44	**44**	44
TNF	***r***	−0.284	−0.095	−0.179	**−0**.**420**	−0.018	**−0**.**382**	−0.201	−0.139	**−0**.**359**
	***p***	0.059	0.535	0.240	**0**.004*	0.904	**0**.010*	0.186	0.361	**0**.016*
	***n***	45	45	45	**45**	45	**45**	45	45	**45**
INF-γ	***r***	−0.065	−0.246	0.003	−0.127	−0.176	−0.049	0.068	−0.048	−0.133
	***p***	0.675	0.108	0.983	0.411	0.252	0.753	0.659	0.757	0.389
	***n***	44	44	44	44	44	44	44	44	44
IL-17A	***r***	0.036	0.214	0.021	−0.071	0.100	−0.047	−0.130	−0.050	0.007
	***p***	0.817	0.159	0.891	0.642	0.512	0.760	0.396	0.746	0.963
	***n***	45	45	45	45	45	45	45	45	45

VF: Verbal Fluency, MBNT: Modified Boston Naming Test, MMSE: Mini-Mental State Examination, WLL: Word List Learning, WLR: Word List Recall, WLRD: Word List Recognition Discriminability, CP: Constructional Praxis, PR: Praxis Recall and CTS: CERAD Total Score.

## Discussion

Our study showed that patients who survived sepsis underperformed on MMSE and constructional praxis 24 h after ICU discharge. After one year, there were improvements in all cognitive tests, except in the constructional praxis. Patients’ performance on the CERAD was influenced by IQCODE, serum levels of NSE and IFN-γ at UCI discharge, and APACHE II, while performance on MMSE was influenced by IL-6 at ICU discharge, time between sepsis diagnosis and antibiotics, haloperidol, mean blood glucose level during ICU stay. Both tests were influenced by level of education and time after ICU discharge.

To the best of our knowledge this is the first study on SAE that employed the CERAD. CERAD has several advantages in comparison to batteries and tests previously used to evaluated global cognitive function in patients with severe sepsis and septic shock, such as CAM-ICU^9–11^, MMSE^12–14^, m-TICS^6,15^. First, CERAD is considered a robust test, being less influenced by language and cultural differences^16–18^. Second, CERAD has been shown to have a single factor structure, supporting our goal to use a global cognition score that could differentiate patients with cognitive impairment and ‘normal’ subjects^19,20^. At last, CERAD had a better predictive capacity than MMSE on the current study, as demonstrated by a greater marginal R^2^, a measure of how well observed outcomes are replicated by the model. Altogether, CERAD seems to be a promising tool for cognitive evaluation in SAE.

In order to strengthen the cognitive assessment, we also applied TMT forms A and B. Nevertheless, TMT did not provide satisfactory information as almost all patients were unable to perform the test within the maximum time both at ICU discharge and at re-evaluation. Accordingly, TMT does not seem an adequate tool for cognitive evaluation of patients with SAE.

Our study found some parameters associated with SAE. Serum concentration of IL-6 at discharge, time after ICU discharge, time between sepsis diagnosis and antibiotic administration, cumulative dose of haloperidol and mean levels of blood glucose during ICU stay affected the performance of these patients on MMSE. Among these variables, attention should be given to blood glucose levels during ICU stay. It is well established that glycemic control in ICU is able to improve outcomes in critically ill patients^21^, and it is also known that glucose and insulin are important modulators of cognitive function^22–24^. Indeed, prevention of hyperglycemia in critically ill patients seems to be a promising neuroprotective strategy capable of preventing both acute cognitive dysfunction as long-term cognitive dysfunction in survivors^25^. To the best of our knowledge this is the first study that showed the relationship between glycemic control and cognitive performance in patients with severe sepsis and septic shock, suggesting an additional benefit of glycemic control as measure with potentially neuroprotective effect.

Haloperidol, a first-generation antipsychotic medication, is primarily a dopamine 2 (D2) receptor antagonist, but it can also block D1 receptors in prefrontal cortex which are critical for cognitive tasks, mainly working memory^26^. This “side effect” may in part explain the detrimental effects of haloperidol on cognition in our study, especially on higher doses^27^.

As discussed before, the CERAD was more robust than the MMSE in our study, but the parameters that influenced the performance of patients on the CERAD revealed to be non-controllable: premorbid cognitive capacity, as measured by IQCODE, NSE and IFN-γ serum levels at ICU discharge, APACHE II score, as well as time after ICU discharge. Accordingly, CERAD exploratory analysis did not add new elements that could be controlled during the stay of the patients at the ICU. Its main usefulness lies in quantifying the cognitive impairment of these patients.

It is noteworthy that the IQCODE score and education level influenced the performance on the CERAD. The concept of ‘brain reserve’ or ‘cognitive reserve’ refers to the individual’s ability to tolerate the age and disease-related changes without developing cognitive signs or symptoms^28^. Our study provides evidence of the importance of cognitive reserve, represented by education and/or IQCODE score in cognitive performance after sepsis.

The role of inflammation on cognition has been demonstrated in experimental studies and there is increasing evidence of this phenomenon in humans^29–32^. The mechanisms by which cytokines affect cognition are not fully elucidated^33^. Systemic inflammation may lead to an adaptive sickness behavior which persists for a few days, but the persistence of this exacerbated neuroinflammatory process can lead to long-standing cognitive impairment rather than mere transient memory disorders^34^. In our study, through exploratory analysis, we found associations between some inflammatory biomarkers and SAE. Our data show association between serum levels of the cytokines IL-4, IL-6, IL-10 and TNF, and cognitive performance of patients with severe sepsis or septic shock. Even in the multivariate models, inflammatory markers continued to influence cognitive performance. More specifically, IFN-γ levels at ICU discharge negatively influenced patient’s performance on CERAD, while IL-6 levels positively affected performance on MMSE. Despite contradictory at the first glance, these data illustrate the complex effect of these mediators on cognition, and it is not surprising that specific molecules could provide both positive and negative effects on cognition depending on the type, intensity and duration of insult suffered by the individual^35^. The measurement of these cytokines at ICU discharge could be regarded as cognitive outcome biomarkers, allowing better planning of cognitive rehabilitation program and maybe a more realistic information regarding the cognitive prognosis.

Previous studies have reported a relationship between NSE, a marker of neuronal damage, and SAE in the presence of sepsis^36,37^. In the clinical practice, NSE has be used as a biomarker along with other parameters to predict the outcome of comatose patients^38^. In line with this, we found that increased levels of NSE at ICU discharge were associated with worse cognitive performance measured by the CERAD.

Our study has several limitations, the main one related to the sample size. Accordingly, there is a potential risk for false-positive and over-fitting model due to the high number of studied ‘variables’. We were not able to validate our findings in an independent dataset which should be carried out to report prediction accuracy. It is worth emphasizing the challenges to recruit – with very well-defined criteria – these type of patients, and to keep their follow-up. In this regard, we also had significant losses on the follow-up. Despite assessing several molecules and cognitive domains, we still performed a limited cognitive and biomarker evaluation. Multicenter clinical trials with larger samples are necessary to confirm the current findings.

## Conclusion

Patients experiencing severe sepsis or septic shock show cognitive impairment that might improve over time. There are several variables that can influence cognitive outcome, such as baseline cognitive reserve as indicated by IQCODE and educational level. Interestingly, some ICU related variables (mean blood glucose and NSE) may also play a role. Future studies must address whether modification of these latter parameters could determine better long term cognitive outcome.

## Patients and Methods

### Study design

This was a single center prospective study to investigate long-term cognitive outcomes in a sample of patients who survived severe sepsis or septic shock. Outcome measures included cognitive performance and psychiatric health collected 24 hours and around one year after discharge from ICU. The study protocol was approved by the local Ethics Committee (0319.0.203.000 – 11) and written informed consent was obtained from all participants or representative prior testing. In addition, all methods were performed in accordance with the relevant guidelines and regulation.

### Inclusion/exclusion criteria

Based on the diagnostic criteria of the 2001 SCCM/ESICM/ACCP/ATS/SIS International Sepsis Definitions Conference^39^, the inclusion criteria were history of severe sepsis (presence of sepsis and concomitant acute organ dysfunction occurring in at least one organ) or septic shock (persistent arterial hypotension unexplained by other causes, despite adequate volume resuscitation) in patients discharged from the ICU at the University Hospital of the Universidade Federal de Minas Gerais, Brazil. The exclusion criteria were age bellow 18 years, pregnancy, any disease with prognosis below three months, immunosuppression, neurological disease present at the time of inclusion (epilepsy, cancer, neuroinfection, stroke, trauma), acute decompensated renal failure or hepatic insufficiency, tracheostomy or any other condition leading to speech incapacity.

### Laboratory work-up

All patients underwent routine diagnostic laboratory tests while at the ICU comprising blood cell count, creatinine, lactate and C reactive protein (CRP). For each patient, we calculated the mean overall glucose level during ICU stay from all glucose values measured. Glucose was obtained from arterial blood samples by means of a handheld glucose measurement devise^40^.

Besides these routine laboratory tests, serum levels of cytokines (IL-2, IL-4, IL-6, IL-10, TNF, IFN-γ, and IL-17A), and sTREM-1, whose expression are up-regulated in the presence of extracellular bacteria and fungi and in inflammatory conditions, were determined. Brain-derived neurotrophic factor (BDNF), the main neurotrophic factor in the central nervous system, and the neuron specific enolase (NSE), a glycolytic protein expressed in neurons and neuroendocrine cells, were also measured.

The measurement of cytokines, sTREM-1, BDNF and NSE was performed at ICU discharge and are detailed as follows. For serum collections, we allowed the blood to clot 15–30 minutes and separated the serum by centrifugation at 1,000–2,000 g at 4 °C for 10 minutes. Serum was aliquoted into separate cryotubes and kept frozen at −80 °C until ready for assay. For the assessment of sTREM-1, BDNF and NSE, samples were run in duplicate using commercial ELISA kits according to the manufacturer’s instructions (R&D Systems, USA and BD Biosciences, USA). The cytokine panel was evaluated through Cytometric Bead Array (CBA) using commercial BD CBA Human Th1/Th2/Th17 Cytokine Kit according to the manufacturer (BD Biosciences, USA). The assessment was performed blind to the clinical status of the subjects.

### Medical record information

Medical data collected were age, gender, level of education, APACHE II score^41^, SOFA score^42^, time of antimicrobial administration after recognition of septic shock or severe sepsis, ICU length of stay, site of infection, microbiological results and comorbid diseases. Midazolam, fentanyl, dobutamine, noradrenaline and haloperidol were set as cumulative dose throughout ICU stay. The treatment of all patients was carried out at the discretion of the ICU assistant physicians^43^.

### Neuropsychological assessment

Since most patients underwent intubation and sedation during their stay in ICU, the first cognitive testing was performed 24 h after ICU discharge. The second cognitive testing was performed around one year after hospital discharge.

Global cognition was assessed using the CERAD^44^, a validated assessment battery that includes measures of verbal fluency, confrontational naming (15-item Boston Naming Test), the Mini-Mental State examination, measures of verbal learning, recall and recognition, and constructional praxis performance and recall. The CERAD battery was originally designed to evaluate patients with Alzheimer’s Disease, but it is also used to measure general cognition in different clinical contexts^45–47^.

Psychomotor speed and divided attention were also assessed using the Trail Making Test (TMT) forms A and B. The TMT form A evaluates visuoperceptual abilities, TMT form B reflects primarily working memory and secondarily task-switching ability, while B minus A provides a relatively accurate index of executive control^48^.

### Estimated premorbid cognitive impairment

Due to the impossibility of obtaining a baseline cognitive performance score for patients with sepsis, premorbid cognitive impairment was estimated based on the Informant Questionnaire on Cognitive Decline in the Elderly (IQCODE)^49^. IQCODE is the most widely-used informant instruments available, and was developed to measuring cognitive decline from a pre-morbid level using informant report. IQCODE has high reliability and measures a single general factor of cognitive decline^50,51^.The questionnaire was applied to informants during the stay of the patient at the hospital.

### Statistics

Data were analyzed using R (version 3.2.2)^52^ and Statistical Package for Social Sciences (V.19.0, SPSS Inc, Chicago, Illinois, USA). Normality of data distributions were evaluated for the study. Student T, Mann-Whitney U, chi-squared tests were used for data analysis when appropriate. The mean score obtained in CERAD battery subtests were compared with expected cutoff point described by Bertolucci *et al*.^16^ using one sample T Test. Difference in cognitive parameters over time, controlling for possible confounding factors, and the influence of the variables on cognitive parameters was calculated by using marginal model. Marginal models also known for GEE method (Generalized Estimating Equations)^53^ can be considered as a generalized linear model^54^ extension which incorporate the expected correlation between the measurements taken in the same individual. Because of its simplicity in interpretation and lack of distributional assumptions, marginal models have been preferred as an extension of the generalized linear models for longitudinal data^55^.

The stepwise regression was used to identify a useful subset of predictors. First a univariate marginal linear model was performed using the forward stepwise method to select variables that correlated with cognitive performance in patients after sepsis. Variables with p < 0.15 were tested for independency in multiple marginal regression analysis using the backward stepwise method. A p-value < 0.05 was considered statistically significant. Variance inflation factor (VIF) was used to assess multicollinearity and Marginal R^2^ values were used to describe model fit.

### Data availability

The datasets generated during and/or analysed during the current study are available from the corresponding author on reasonable request.

## Electronic supplementary material

Supplementary Table S1
