# Supplementary material for: Factors Associated With Short and Long Term Cognitive Changes in Patients With Sepsis
Source: Sci Rep. 2018 Mar 14;8:4509. doi: 10.1038/s41598-018-22754-3 (PMC5852240; doi:10.1038/s41598-018-22754-3)
Supplement: Supplementary file 1 — Supplementary Table S1 [file 41598_2018_22754_MOESM1_ESM.docx]

**Supplemental Material**

**Factors associated with short and long term cognitive changes in patients with sepsis**

Allan J. C. Calsavara^1, 2^, Priscila A. Costa^1^, Vandack Nobre^2,3^, Antonio L. Teixeira^2,4^

^1^ School of Medicine, Universidade Federal de Ouro Preto, Ouro Preto, MG, Brazil;

^2^ Postgraduate Program in Health Sciences: Infectious Diseases and Tropical Medicine, School of Medicine, Universidade Federal de Minas Gerais, Belo Horizonte, MG, Brazil.

^3^ Núcleo Interdisciplinar de Investigação em Medicina Intensiva - NIIMI

^4^ Neuropsychiatry Program & Immuno-Psychiatry Lab, Department of Psychiatry and Behavioral Sciences, McGovern Medical School, University of Texas Health Science Center at Houston, Houston, TX, USA.

Correspondence and requests for materials should be addressed to A. J. C. C. (email: allancalsavara@medicina.ufop.br

Table of Contents: Page

| Supplementary Table S1 | Comparison of demographic and clinical characteristics between patients re-evaluated and not re-evaluated | 2 |
| --- | --- | --- |

Supplementary Table S1. Comparison of demographic and clinical characteristics between patients re-evaluated and not re-evaluated after one year

|  | Re-evaluated | | No vs. Yes  P value |
| --- | --- | --- | --- |
|  | **No (n=17)** | **Yes (n-=16)** |  |
| Age at sepsis (years) | 48.88 ± 16.28 | 49.19 ± 14.42 | 0.955 |
| Education (years) | 7 (4.5 – 9.0) | 5.5 (8.8 – 4.0) | 0.637 |
| APACHE II Score | 16.7 ± 6.1 | 16.2 ± 8.4 | 0.840 |
| SOFA Score at ICU admission | 6 (4.5 - 9) | 7.0 (4.3 – 9.0) | 0.928 |
| Administration of antimicrobial after recognition of septic shock or severe sepsis (hours) | 1.8 (1.3 – 6.6) | 4.9 (1.9 – 19.2) | 0.121 |
| Laboratory at admission in ICU |  |  |  |
| Lactate (mmol/L) | 1.65 (1.1 – 2.9) | 2.1 (2.7-1.0) | 0.884 |
| CRP (mg/dL) | 275.4± 124.7 | 186.9 ± 119.6 | 0.05 |
| Mean blood glucose during stay in ICU (mg/dL) | 136.9 ± 44.4 | 133.0 ± 27.7 | 0.764 |
| Septic shock | 13 (76.5) | 12 (75.0) | 0.922 |
| Sites of infection |  |  |  |
| Lung | 4 (23.5) | 7 (43.8%) | 0.218 |
| Intra-abdominal | 3 (17.6) | 3 (18.8) | 0.935 |
| Urinary tract | 4 (23.5) | 1 (6.3) | 0.335 |
| Catheter | 2 (12.5) | 3 (17.6) | 1.000 |
| Skin | 2 (11.8) | 0 (0.0) | 0.485 |
| Unknown | 1 (5.9) | 3 (18.8) | 0.335 |
| Positive blood culture | 8 (47.1) | 5 (31.3) | 0.353 |
| Comorbid diseases |  |  |  |
| Congestive heart failure | 2 (11.8) | 1 (6.3) | 1.000 |
| Chronic renal failure | 3 (17.6) | 3 (18.8) | 1.000 |
| COPD | 0 (0.0) | 1 (6.3) | 1.000 |
| Arterial hypertension | 9 (56.3) | 6 (37.5) | 0.288 |
| Diabetes mellitus | 5 (29.4) | 4 (25.0) | 1.000 |
| Need for noradrenaline in the first 72h | 12 (70.6) | 12 (75.0) | 1.000 |
| Need for dobutamine in the first 72h | 2 (11.8) | 3 (18.8) | 0.656 |
| Drug used in ICU |  |  |  |
| Midazolam | 6 (35.3) | 7 (43.8) | 0.619 |
| Fentanyl | 8 (47.1) | 8 (50.0) | 0.866 |
| Noradrenaline | 12 (70.6) | 14 (87.5) | 0.398 |
| Dobutamine | 2 (11.8) | 5 (31.3) | 0.225 |
| Haloperidol | 4 (23.5) | 6 (37.5) | 0.465 |
| Cumulative dose |  |  |  |
| Midazolam (mg) | 270.0 (130.0 – 1117.8)) | 180.0 (40.0 – 1440.0) | 0.616 |
| Fentanyl (μg) | 4635.0 (620.0 – 25540.0) | 2720.0 (606.0 - 9345.0) | 0.674 |
| Noradrenaline (mg) | 36.7 (8.65 – 60.63) | 34.5 (9.28 – 73.0) | 0.877 |
| Dobutamine (mg) | 2648.6 (1161.2 – 4136.0) | 716.0 (391.5 – 2479.0) | 0.245 |
| Haloperidol (mg) | 12.5 (6.25 – 118.13) | 3.5 (2.5 – 26.7) | 0.195 |
| Need for corticosteroids in the first 72h | 5 (29.4) | 1 (6.3) | 0.175 |
| Need for mechanical ventilation in the first 72h | 12 (70.6) | 8 (50.0) | 0.226 |
| Need for hemodialysis in the first 72h | 0 (0.0) | 3 (18.8) | 0.103 |
| CERAD Score | 49.76 ± 16.94 | 51.31 ± 14.39 | 0.780 |
| IQCODE | 3.12 (3.00 – 3.35) | 3.10 (3.00 – 3.50) | 0.785 |
| Data presented as mean ± SD, N (%) or median (IQR25-75). | |  |  |
